# Community knowledge and perceptions about indoor residual spraying for malaria prevention in Soroti district, Uganda: a cross-sectional study

**DOI:** 10.1186/1475-2875-12-170

**Published:** 2013-05-27

**Authors:** Michael Ediau, Juliet N Babirye, Nazarius M Tumwesigye, Joseph KB Matovu, Simba Machingaidze, Olico Okui, Rhoda K Wanyenze, Peter Waiswa

**Affiliations:** 1 Makerere University School of Public Health, PO Box 7072, Kampala, Uganda; 2 Division of Global Health, Karolinska Institutet, Stockholm, Sweden; 3 ChildFund Uganda, PO Box 3341, Kampala, Uganda

**Keywords:** Indoor residual spraying, Community, Knowledge, Perceptions, Malaria prevention

## Abstract

**Background:**

Malaria is the leading cause of morbidity and mortality in Uganda. The Ministry of Health (MoH) plans to scale up indoor residual spraying (IRS) for malaria vector control. However, there is limited information on community knowledge and perceptions towards IRS. This study assessed community knowledge and perceptions about IRS in Soroti district, eastern Uganda.

**Methods:**

The study was cross-sectional and it covered 770 randomly selected households in urban and rural settings in Soroti district, Eastern Uganda. The respondents were heads of household and or their proxies. The data were collected on the sociodemographic characteristics, knowledge of the insecticides that could be used for IRS, parts of the houses that would be sprayed, importance of IRS, role of household heads in IRS programme, frequency and the time of spraying. Responses to the questions on these areas were used to create a composite dependent variable categorized as knowledgeable if they had responded correctly to at least three questions or not knowledgeable about IRS if they responded correctly to less than three questions. In addition, respondents were asked if they thought the IRS programme would be beneficial or not. Bivariate and multivariate logistic regression analyses were carried out using SPSS version 17.

**Results:**

Less than half, (48.6%, 374/770) of the respondents were knowledgeable about IRS. Urban residents (AOR 1.92, 95% CI 1.04-3.56) and those with secondary education or higher (AOR 4.81, 95% CI 2.72-8.52) were knowledgeable about IRS. Three-quarters, (74.4%, 354/473) of respondents who had ever heard of IRS, perceived it as beneficial. Two-thirds, (66.4%, 314/473) reported that IRS would have negative effects. Respondents who reported that, IRS programme is beneficial were: 23 years or older (AOR 2.17, 95% CI 1.07-4.38), had attained secondary education or higher (AOR 2.16, 95% CI 1.22-3.83) and were knowledgeable about IRS (AOR 2.21, 95% CI 1.17-4.17).

**Conclusions:**

Knowledge about IRS is inadequate and negative perceptions about its use are prominent especially among the rural and less educated individuals. To ensure householders’ cooperation and participation in the IRS programme, adequate community mobilization and sensitization is needed prior to use of IRS for effective malaria control.

## Background

In developing countries, malaria still causes the highest morbidity and mortality. In 2010, the World Health Organization (WHO) estimated that 3.3 billion people were at risk of getting malaria, 216 million developed malaria and about 700,000 of them died. Most (86%) of the victims were children under five years of age, and over 91% of malaria deaths occurred in Africa [1].

In Uganda, stable and perennial malaria transmission occurs in 90 to 95 percent of the country. In the rest of the country, particularly in the highland areas, there is low and unstable transmission, with potential for epidemics [2]. Malaria remains the leading cause of mortality in Uganda. It is responsible for 21% (including 27% of under-five) of all hospital deaths [3]. Malaria is also known to be a significant cause of morbidity and mortality in pregnant women in Uganda [3]. The high contribution of malaria to under-five and maternal mortality significantly hampers Uganda’s progress towards achievement of the Millennium Development Goal (MDG) 4, that is, to reduce by two-thirds, between 1990 and 2015, the under-five mortality rate as well as MGD 5 that is to reduce by three quarters, between 1990 and 2015, the maternal mortality ratio. Progress achievement of MDGs 4, 5 as well as target 6C of MDG 6 that is to have halted by 2015 and begun to reverse the incidence of malaria and other major diseases has been reported to be too slow [4]. This calls for implementation of effective and sustainable malaria control measures in order to reduce the burden of malaria. Vector control, especially indoor residual spraying (IRS) remains one of the most effective methods for preventing malaria transmission [5].

WHO recommends IRS, with dichlorodiphenyltrichloroethane (DDT), as a malaria vector control measure [6]. Following this recommendation, the Ministry of Health (MoH) introduced IRS as one of its malaria control strategies and more specifically a key component of the vector control intervention strategy [7]. MoH planned to cover at least 80% of all targeted structures in areas of unstable transmission of malaria countrywide by end of the year 2010 [7]. However, by the year 2011, only 7.2% had been sprayed with insecticides in the last 12 months [8]. MoH has since revised this target to 30% of targeted households sprayed in the last 12 months by 2015 [9]. However, community knowledge and perceptions about house spraying have been found to be critical for the IRS programme to be successful [10].

Previous studies show that community understanding of and beliefs about the purpose of an IRS programme varied but with less importance being attached to malaria transmission prevention [11-14]. Other studies have demonstrated that communities have positive expectations when IRS or related prevention interventions are introduced [12-16]. However, they may have fears and concerns about IRS programmes, which may lead to refusal of IRS [13,15-17]. Therefore, addressing community concerns about IRS and ensuring that misperceptions are corrected ensures responsiveness to community needs and increases uptake of IRS interventions [12-16].

Understanding of the function of the IRS programme has been related to community compliance with the programme [14,16]. Spraying coverage also depends on whether members of households perceive the IRS programme intervention as beneficial, in terms of how effective the insecticide is against mosquitoes and other nuisance insects, as well as the number and intensity of unwanted side effects [14,18]. It is thus necessary to understand community beliefs and knowledge when planning or evaluating vector control activities [11].

While by the time of this study the Ministry of Health in conjunction with other partners had started implementing a pilot IRS programme in one of the districts (Katakwi district) which neighbors Soroti, in Soroti district itself, no IRS programme had been conducted nor were communities mobilized and sensitized about the upcoming IRS programme for malaria control. At the same time, no information was available on communities’ knowledge and perceptions about IRS. The purpose of this study was therefore to assess community knowledge and perceptions about IRS in Soroti district, north-eastern Uganda.

## Methods

### Study area

The study was conducted in Soroti district, located in north-eastern Uganda about 300 km from Kampala, the capital city. At the time of the study, the district was administratively divided into three counties (Soroti, Serere and Kasilo counties) and one Municipality (Soroti Municipality). The district had 14 subcounties and three divisions (in Soroti Municipality). Health services are provided by one regional referral hospital, four health centres IV, 20 health centres III and 22 health centres II. Malaria is the leading cause of morbidity and mortality in Soroti, contributing to 23% of the total disease burden (Unpublished Soroti District Health Sector Annual Report, 2007/2008).

### Study design and sampling procedure

**Figure 1:**
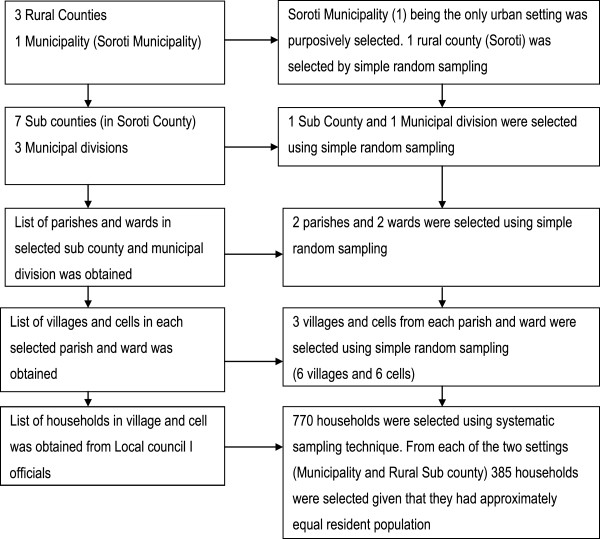
Schematic flow of sampling procedure.

A household survey among 770 household heads and or their proxies was conducted from February to March 2009. The sample was estimated with a 95% confidence interval (CI), a precision of 5%, with an estimated proportion of household heads who had heard of IRS of 50% and a design effect of 2.0 to cater for intra-cluster variability. Only adults aged 18 years and above were included in the study. In selection of the respondents, a multistage sampling technique was employed. A table of random numbers was used at each stage of the sampling process from county/municipal council to village/cell level. At the first stage, one county (Soroti county) was selected from three rural counties. Soroti municipal council was purposively selected since it was the only urban setting in the district. At the second stage, one division from Soroti Municipality and one subcounty from Soroti county were selected. At the third stage, two rural from the subcounty selected at the second stage and two urban wards from the division selected from Soroti Municipality were selected. At the fourth stage, six villages from the two rural parishes and six cells from the two urban wards were selected. The total number of households in all selected villages and cells was obtained from the subcounty and municipal division offices. The number of households to be visited in each village/cell was determined by dividing the village size by the total population in the selected three villages and three cells, then multiply by 770 which was the overall sample size. Households that were included in the study were selected using systematic sampling technique, by obtaining a list of all households in a village or cell from the local council officials. A sampling interval was computed by dividing the total number of households in the village by the required sample of households for the study in that village. At the outset, one household was randomly selected from the list of households. Subsequently, every ith household was selected from the list until the sample size for the village/cell was achieved. If the selected household had no eligible individuals, the neighbouring house to the east was considered. If the household head was not available for interview, the spouse or any other household member aged 18 years and above was interviewed (see Figure 1).

### Data management and analysis

Ten Research Assistants, administered a semistructured questionnaire to collect data on the sociodemographic characteristics such as sex, age, education level attained, occupation, marital status, place of residence (rural/urban). Other data collected included: knowledge of the type of insecticides that could be used for IRS, the parts of the house that are sprayed with these insecticides, importance of IRS, role of household heads in ensuring the success of an IRS programme, knowledge on frequency of spraying and the time of spraying. Responses to these questions were used to create a composite variable categorized as knowledgeable or not knowledgeable about IRS. The researchers developed a checklist consisting of a set of questions. The questions and the correctness of answers were derived from MoH and WHO indoor residual spraying guidelines [6,19,20]. Respondents were considered knowledgeable if they had responded correctly [6,19,20] to at least three questions. They were categorized as not knowledgeable about IRS if they responded correctly to less than three questions. Data on the source of IRS information as well as the preferred source of health education were also collected.

Regarding perceptions, respondents were asked if they thought the IRS programme would be beneficial or not when conducted. They were further asked about the perceived benefit as well as negative effects of the IRS programme. Regardless of whether respondents mentioned perceived benefits or not, they were still asked about the perceived negative effects of the IRS programme. In addition, during data collection interviews, Research Assistants explained to all respondents that IRS requires that some household property should be moved out of the houses before house spraying and only returned in the houses after a specified period of time following completion of house spraying. Respondent were also informed that spray teams are required to enter houses in order to spay targeted inner surfaces like walls. Data on respondents’ perceptions about spraying teams entering houses, as well as the requirement to move out property prior to spraying, was also collected.

All data were edited, coded, entered and analysed using SPSS statistical software (version 17). Descriptive statistics were generated on the characteristics and responses of the population. At bivariate and multivariate analyses, variables with a p-value <0.05 were considered statistically significant. Unadjusted and adjusted odds ratios (OR) at 95% CI were used to measure associations. All variables that were statistically significant and all those that were biologically plausible at the bivariate level were entered into a forward stepwise (conditional) logistic regression to identify independent predictors of being knowledgeable about IRS as well as having a positive perception towards the IRS programme. Data quality was ensured through training of research assistants who were fluent in the local language. The data collection tools were also pretested. Meetings between the principal investigator and research assistants were held on a daily basis after data collection to check for completeness of data.

### Ethical considerations

Ethical clearance was obtained from Makerere University School of Public Health Higher Degrees Research and Ethics Committee and independently from the Uganda National Council of Science and Technology.

## Results

### Background characteristics of the respondents

Of the 770 respondents enrolled in this study 50% (385) lived in urban areas, 52.6% (405) were females, 80.3% (618) were aged ≥23 years, 47.7% (367) were peasant farmers, 52.2% (402) had attained education up to primary level, 58.6% (451) were married, while 47.3% (364) had a child who was less than five years old in the household and 63.1% (486) were Iteso.

### Respondents’ knowledge about indoor residual spraying

More than half (61.4%, 473/770) of respondents had heard of IRS. The majority (90.1%, 426/473) of these mentioned that insecticides will be used for IRS. Out of those who mentioned insecticides, 64.3% (274/426) specifically mentioned DDT and 12.9% (55/426) mentioned lambda-cyhalothrin (ICON) as the chemicals/insecticide used for IRS and 32.4% (138/426) said they did not know. Overall 67.8% (289/426) mentioned DDT and/or ICON as the chemicals used in IRS. Regarding the exact parts of the house to be sprayed with the insecticides, 74.0% (350/473) mentioned the different surfaces of inner walls and 24.3% (115/473) said they did not know. Regarding the importance of IRS, 92.4% (437/473) of respondents said that IRS is important because it will help to kill mosquitoes, 62.2% (295/473) reported that it will help to kill other domestic insects, 11.4% (54/473) said it will help in killing rodents, and 5.1% (24/473) reported that they did not know its importance. Reported roles of household heads in IRS exercise included: removing some of the household items from the house prior to spraying (33.0%, 156/473), removing people from the houses prior to spraying (6.3%, 30/473), ensuring that people stayed out of the house during and after spraying (2.7%, 13/473), provide spray team with clean water for mixing chemicals (24.1%, 114/473), 64.5% (305/473) said they did know their roles. Respondents who had ever heard of IRS were asked about the frequency and timing (day or night) of IRS, about one-fifth (15.6%, 74/473) of respondents reported that IRS will be conducted after every three months, 73.4% (347/473) said they did not know the frequency of IRS. Regarding the time of spraying, 25.5% (120/473) reported that the spraying will be done during the day and 61.9% (293/473) said they did not know. These responses were computed into a composite variable and about half (48.6%, 374/770) of respondents were knowledgeable about IRS (see Table 1).

**Table 1:** Respondents’ knowledge of indoor residual spraying

**Variable**	**Frequency**	**Percentage (%)**
**Ever heard of IRS**	**n = 770**	
Yes	473	61.4
No	297	38.6
**Insecticides employed for IRS**	**n = 426**	
DDT	274	64.3
ICON	55	12.9
Don’t know	138	32.4
**The exact parts of the house to be sprayed during IRS**	**n = 473**	
On the surfaces of inner walls	350	74.0
On the surfaces of outer walls	52	11.0
On the inner surfaces of the roof	95	20.1
Don’t know	115	24.3
**Importance of IRS (multiple responses accepted)**	**n = 473**	
To kill mosquitoes	437	92.4
To kill other domestic insects	295	62.2
To kill rodents	54	11.4
Don’t know the importance of IRS	24	5.1
**Roles of household heads in IRS (multiple responses accepted)**	**n = 473**	
Removing some of the household items from the house prior to spraying	156	33.0
Removing people from the house prior to spraying	30	6.3
To ensure that people stay out of the house during and after spraying (for at least 2 hours)	13	2.7
Provide spray team with clean water for mixing chemicals	114	24.1
Don’t know	305	64.5
**Frequency of spraying (multiple responses accepted)**	**n = 473**	
Once	29	6.1
After every three months	74	15.6
After every six months	30	6.3
Annually	8	1.8
Don’t know	347	73.4
**Time of spraying**	**n = 473**	
In the morning hours	56	11.8
During day	120	25.4
At night	9	1.9
Don’t know the time of spraying	293	61.9
**Composite level of knowledge about IRS**		
Knowledgeable	374	48.6
Not knowledgeable	396	51.4

Those that mentioned DDT as a chemical for IRS were mostly urban residents (59.5%, 163/274), 62.0% (170/274) were males and 79.6% (218/274) had attained secondary education and above. Reported sources of information on IRS among respondents included: radio (66.6%, 315/473), health workers (17.1%, 81/473), community members (14.8%, 70/473), local leaders (14.8%, 70/473) and information, education and communication print materials such as posters (3.4%, 16/473).

### Perceptions of indoor residual spraying

This study also examined respondents’ perceptions about the IRS programme if conducted in their area. Of those who had heard of IRS, 74.4% (352/473) reported that IRS will be beneficial while 16.1% (76/473) said IRS will not be beneficial. A small proportion (9.5%, 45/473) said they did not know whether IRS will be beneficial or not while 66.4% (314/473) had a perception that IRS would lead to negative effects.

**Figure 2:**
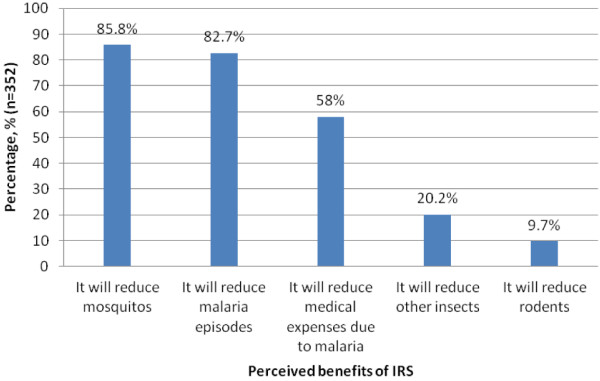
Perceived benefits of IRS programme.

Respondents’ perceptions about IRS varied from perceived benefits to perceived negative effects. The commonest perceived benefits of IRS were: reduction of mosquitoes (85.8%, 302/352) and malaria episodes (82.7%, 291/352) (see Figure 2). Regarding perceived negative effects, most respondents (84.4%, 265/314) thought that IRS might lead to negative health effects, such as cancers and respiratory tract infections. Most respondents (77.7%, 244/314) said the chemicals used for IRS will pollute the environment or contaminate food in the houses (33.1%, 104/314).

### Perceptions about spraying teams entering houses as well as requirement to move some property out prior to spraying

After explaining what IRS entails 79.1% (609/770) respondents reported that they would let spraying teams enter their houses for purposes of conducting IRS and 20.9% (161/770) would not. Most 76.1% (586/770) of the respondents said they would move some of their property out of the house as required prior to spraying while 23.9% (184/770) said they would not. Respondents who were unwilling to move property out of the house prior to spraying cited several reasons including: interference with privacy 96.2% (177/184), interference with security of household property 90.2% (166/184) and also belief that the IRS is a tiresome process 13.6% (25/184).

### Characteristics of those that were knowledgeable and those that had a positive perception about indoor residual spraying

Being knowledgeable about IRS was positively associated with respondents’ residence in urban area (AOR 1.92, 95% CI 1.04-3.56), secondary or higher level of education (AOR 4.81, 95% CI 2.72-8.52) and sources of information about IRS: radio (AOR 2.94, 95% CI 1.60-5.40) and health workers (AOR 5.47, 95% CI 1.89-15.83). Respondents who reported fellow community members (peers) as their source of information on IRS were less likely to be knowledgeable (AOR 0.40, 95% CI 0.20-0.81, Table 2).

**Table 2:** Factors associated with being knowledgeable about IRS as well as perception that IRS will be a beneficial intervention

**Variable**	**Knowledgeable about IRS**		**Perception that IRS will be beneficial**	
	**Unadj. OR (95%CI)**	**Adj OR (95% CI)**	**Unadj. OR (95%CI)**	**Adj OR (95% CI)**
**Residence**				
Urban	2.04 (1.53-2.72)	1.92 (1.04-3.56)*		
Rural	1.0	1.0		
**Sex**				
Male	2.17 (1.63-2.90)	1.33 (0.77-2.30)		
Female	1.0	1.0		
**Age in years**				
18 – 22	1.0	1.0	1.0	1.0
≥ 23	2.61 (1.78-3.81)	1.92 (0.93-3.94)	2.03 (1.05-3.92)	2.17 (1.07-4.38)*
**Occupation**				
Peasant farmer	0.31 (0.23-0.41)	0.54 (0.29-1.01)		
Other	1.0	1.0		
**Highest education level**				
Primary and below	1.0	1.0	1.0	1.0
Secondary or beyond	10.13 (7.27-14.11)	4.81 (2.72-8.52)*	2.43 (1.46-4.02)	2.16 (1.22-3.83)*
**Source of IRS information**				
Radio				
Yes	3.77 (2.38-5.96)	2.94 (1.60-5.40)*	2.17 (1.30-3.62)	1.44 (0.80-2.60)
No	1.0	1.0	1.0	1.0
Health workers				
Yes	4.86 (1.91-12.37)	5.47 (1.89-15.83)*		
No	1.0	1.0		
Peers (community members)				
Yes	1.98 (0.95-4.13)	0.40 (0.20-0.81)*	0.38 (0.22-0.67)*	0.44 (0.24-0.84)*
No	1.0	1.0	1.0	1.0
Local leaders				
Yes	0.31 (0.18-0.53)	2.10 (0.88-4.94)	0.39 (0.22-0.67)	1.21 (0.50-2.91)
No	1.0	1.0	1.0	1.0
**Knowledge level about IRS**				
Knowledgeable			3.67 (2.09-6.44)	2.21 (1.17-4.17)*
Not Knowledgeable			1.0	1.0

* Statistically significant association.

Independent predictors of having a positive perception about the IRS programme included: age ≥23 years (AOR 2.17, 95% CI 1.07-4.38), having attained secondary or higher level of education (AOR 2.16, 95% CI 1.22-3.83), and being knowledgeable about IRS (AOR 2.48, 95% CI 1.33-4.61). Respondents who mentioned fellow community members as their source of information on IRS were not likely to perceive IRS as being a beneficial programme (AOR 0.44, 95% CI 0.24-0.84, Table 2).

### Preferred sources of health education on indoor residual spraying

Reported preferred sources of health education on IRS were: community health workers (80.0%, 616/770), radio (79.2%, 619/770), health workers (78.1%, 601/770), religious leaders in gatherings such as churches (17.9%, 138/770), printed information, education and communication materials (12.9%, 99/770), community leaders (10.6%, 82/770) and television (1.8%, 14/770).

## Discussion

This study found significant knowledge gaps about IRS as well as negative and positive perceptions about its use among the communities in rural Uganda, with over 51% of the respondents falling in the not knowledgeable category.

About two-thirds of the study respondents had heard of IRS. Almost all respondents who had heard about IRS (92.4%) knew its importance in reducing mosquitoes and malaria. Overall, 48.6% of respondents were graded as knowledgeable about IRS according to this study. Prior to this study, mass community mobilization and sensitization campaigns for IRS were conducted in a neighbouring district, which may explain the significant level of knowledge, especially in the urban areas. Given that the IRS programme promotion campaigns had not yet been conducted in Soroti District, these findings give a big impetus for the IRS programme in the future.

A large proportion of the study respondents had a positive perception towards IRS. This was consistent with findings elsewhere that showed positive community expectations when IRS or related prevention interventions were introduced [12-16]. This study also found that despite having positive perceptions towards IRS, a large proportion still had negative perceptions towards its use. These negative perceptions and the limited knowledge were more prevalent in rural areas, which have the greatest need for effective malaria control strategies [13-17]. This, therefore, calls for special IRS promotion efforts and strategies that target such rural communities [10,18].

The most known insecticide/chemical for IRS was DDT. This was a surprising finding given that ICON (and not DDT) was the chemical used for IRS in the districts bordering the study area as well as other parts of the country. This misinformation may be attributed to multiple sources of information, including the media, since there have been several debates on the use of DDT in the local media. Anti-DDT activists have been emphasizing the negative effects of DDT, which may have negatively affected community perceptions of IRS, even when ICON or other less controversial chemicals are used. These negative perceptions could potentially affect the coverage of the IRS programme. In India, the causes of refusal for IRS varied depending on the insecticide sprayed. Since most vector control was based on DDT indoor spraying, the general opinion was against the usefulness of this insecticide, thus the coverage was poor [21]. According to WHO there is no justification for preventing the use of DDT for IRS provided a clear national policy and adequate safeguards for storage, transport and disposal are in place and that WHO recommendations are adhered to [10]. Future IRS programmes need to sensitize communities on the chemical used as this influences acceptability and uptake.

Urban respondents were about twice more likely to be knowledgeable about IRS than their counterparts in the rural setting. There are several possible explanations for this. People in urban settings tend to have more access to information through mass media, such as radio, television and health promotion campaigns. A recent demographic health survey indicated that 75% of urban households in Uganda own a radio, compared to 58% of rural households. Radio is an important source of health information in Uganda and most respondents in this study cited radio as their source of information. Urban residents are also likely to be more educated and therefore able to quickly access information about IRS as compared to those in the rural areas [8]. Correspondingly, this study found that higher education level is a predictor for being knowledgeable about IRS. Health workers were the other significant source of information about IRS, but community members or peers as sources of information were negatively associated with being knowledgeable about IRS, since mass mobilization and education in these communities had not commenced.

The most prominent perceived benefit of IRS was reduction of nuisances of mosquitoes, cited by 85% of the respondents. As reported elsewhere, this finding seems to indicate that participants were more concerned about the mosquitoes than malaria as a disease [13]. Thus designers of information, education and communication messages need to package the benefits of IRS as a mosquito bite reduction initiative but also emphasize its role as a malaria control strategy [12].

### Study limitations

This study was conducted in a community that had not yet been directly mobilized or educated about IRS, nor did it explore actual experiences with use of IRS. As such, this particular study may not be able to differentiate between the real experiences and anticipated fears of IRS use. Nevertheless, the study highlights significant community concerns about IRS that should be anticipated and addressed through health education in order to ensure success of such a programme. This study did not use qualitative data collection methods like focus groups discussions which would have enabled us to further explore the community perceptions about IRS.

## Conclusions

The study found that prior to IRS implementation, knowledge about IRS was inadequate and more so knowledge about the roles of household heads in the IRS programme was evidently limited. Although the majority of respondents had positive perceptions, a large proportion still had negative perceptions towards the use of IRS. Negative perceptions about IRS use as well as limited knowledge were prominent especially among the rural and less educated individuals. Therefore to ensure householders’ cooperation and participation in the IRS processes in order to achieve a successful IRS programme, adequate community mobilization and sensitization is needed, prior to introduction of IRS to address the identified knowledge gaps and poor perceptions about it.

## Competing interests

The authors declare that they have no competing interests.

## Authors’ contributions

ME designed the study, coordinated recruitment of participants, collected and entered data, analysed data and drafted the manuscript. JNB, PW, and OO designed the study, participated in data analysis and reviewed the draft manuscript. NMT guided data analysis and reviewed the draft manuscript. RKW, JM and SM reviewed the draft manuscript. All authors read and approved the final manuscript.
